# Methicillin-Sensitive Staphylococcus aureus (MSSA) Bacteremia With Concomitant Prostatic and Panspinal Epidural Abscesses and Cerebral Infarcts: A Rare Presentation

**DOI:** 10.7759/cureus.111831

**Published:** 2026-06-30

**Authors:** Muhammad Usman, Abdul Basit, Muhammad A Usman, Abdullah Saeed, Shahzaib Khaliq, Laiba Murtaza, Muhammad Zatmar Khan, Zarmina Bashir

**Affiliations:** 1 Internal Medicine, Mercy Hospital Fort Smith, Fort Smith, USA; 2 Internal Medicine, Arkansas College of Osteopathic Medicine, Fort Smith, USA; 3 Internal Medicine, King Edward Medical University, Lahore, PAK; 4 Internal medicine, King Edward Medical University, Lahore, PAK; 5 Internal Medicine, Baptist Health System, Fort Smith, USA; 6 Internal Medicine, Khyber Medical College, Peshawar, PAK

**Keywords:** antimicrobial therapy, panspinal epidural abscess, persistent mssa bacteremia, prostate abscess, supportive and palliative care

## Abstract

Methicillin-sensitive *Staphylococcus aureus* (MSSA) bacteremia is a severe bloodstream infection with potential for metastatic spread to multiple organs. Although prostatic abscesses are typically associated with gram-negative organisms, MSSA may rarely cause disseminated multisystem infection, particularly in patients with significant comorbidities. Early recognition and aggressive intervention are essential to improve outcomes. A 64-year-old man with diabetes, hypertension, and peripheral arterial disease presented with back pain and new-onset atrial fibrillation. Blood cultures confirmed MSSA bacteremia. Imaging revealed a prostatic abscess, extensive panspinal epidural abscess with discitis, and multiple septic cerebral infarcts. After unsuccessful percutaneous drainage, emergent neurosurgical decompression was performed. Antimicrobial therapy was tailored to optimize CNS penetration and address high bacterial burden. The hospital course was complicated by renal failure requiring hemodialysis, nosocomial infections, and extensive venous thromboses. This case underscores the aggressive nature of disseminated MSSA bacteremia and the importance of multidisciplinary management.

## Introduction

*Staphylococcus aureus* is the leading cause of bacteremia, with an incidence ranging from 9.3 to 65 cases/100,000/year [1]. *Staphylococcus aureus* bacteremia is notorious for seeding multiple organs, leading to metastatic infections in a large fraction of patients. In fact, one study demonstrated that roughly one-third of patients with methicillin-sensitive *Staphylococcus aureus* (MSSA) bacteremia develop infectious foci [2]. Metastatic infections are serious complications that need prompt evaluation; failure to identify these may lead to relapse of MSSA bacteremia and poor prognosis [3]. Most observed foci of metastatic infections include endocarditis (12%), septic arthritis (7%), vertebral osteomyelitis (4%), splenic abscess, psoas abscess, and septic pulmonary emboli [2]. However, metastatic foci in the brain, epidural space, and prostate are quite rare. Risk factors for MSSA bacteremia include implantable indwelling catheters, recent surgical procedures, IV drug abuse, and diabetes mellitus. Ninety-day mortality is 39% in patients with prolonged MSSA infection (more than 48 hours) [4].

The choice of antibiotics for MSSA bacteremia depends on regional antibiotic resistance trends. Regions with MRSA rates below 5% may initiate beta-lactam antibiotics such as nafcillin, flucloxacillin, oxacillin, benzylpenicillin, cloxacillin, or cefazolin [5]. However, a meta-analysis indicates that cefazolin is associated with lower nephrotoxicity and 30-day mortality compared with other anti-staphylococcal penicillins [6].

We present here a case of MSSA bacteremia complicated by an unusually extensive constellation of abscesses, including a prostatic abscess and a panspinal epidural abscess/discitis extending from the cervical through the thoracolumbar spine, along with multiple septic infarcts in the brain. Prostatic abscesses are usually caused by gram-negative organisms such as *Escherichia coli*, whereas those caused by *Staphylococcus aureus* are infrequently reported.

## Case presentation

A 64-year-old man with a past medical history significant for diabetes mellitus, hypertension, and peripheral arterial disease s/p bilateral femoral-to-popliteal artery bypass presented to the hospital with low back pain and new-onset atrial fibrillation. The patient reported no urinary symptoms or fevers and varying-intensity back pain over the past two days. Physical examination at the time of presentation was completely unremarkable, including normal motor strength, sensory examination, intact reflexes, no bowel/bladder dysfunction, and no signs of myelopathy. Vital signs were notable for tachycardia with an irregular rhythm and no fever. Laboratory evaluations are summarized in Table 1.

**Table 1:** Initial laboratory evaluation

Test name	Result	Reference range
Red blood cell count	4.63 million/µL	4.63-6.08 million/µL
Hemoglobin	12.7 g/dL	13.7-17.5 g/dL
Hematocrit	42.4%	40.1-51.0%
Platelet count	216 thousand/µL	163-337 thousand/µL
Mean platelet volume	12.9 fL	9.4-12.4 fL
Neutrophils	85%	34-71%
Lymphocytes	8%	19-53%
Monocytes	5%	5-13%
Eosinophils	0%	1-7%
Basophils	0%	0-1%
Immature granulocytes	2%	0-1%
Sodium	129 mmol/L	136-145 mmol/L
Potassium	3.6 mmol/L	3.5-5.1 mmol/L
Chloride	99 mmol/L	98-107 mmol/L
Carbon dioxide (bicarbonate)	16 mmol/L	22-29 mmol/L
Calcium	7.7 mg/dL	8.8-10.2 mg/dL
Blood urea nitrogen	26 mg/dL	8-23 mg/dL
Creatinine	0.90 mg/dL	0.67-1.17 mg/dL
Glucose	248 mg/dL	74-99 mg/dL
Total protein	4.7 g/dL	6.4-8.3 g/dL
Albumin	2.6 g/dL	4.0-4.9 g/dL
Total bilirubin	0.4 mg/dL	0.0-1.2 mg/dL
Alkaline phosphatase	67 U/L	40-129 U/L
Aspartate aminotransferase	10 U/L	0-40 U/L
Alanine aminotransferase	11 U/L	≤41 U/L
Estimated glomerular filtration rate	>60 mL/min/1.73 m²	≥60 mL/min/1.73 m²
Specific gravity	1.043	1.003-1.035
Urine pH	6.5	5.0-8.0
Leukocyte esterase (urine)	Trace	Negative
Nitrite (urine)	Negative	Negative
Protein (urine)	1+	Negative
Glucose (urine)	1+	Negative
Ketones (urine)	Trace	Negative
Blood	Negative	Negative
White blood cells	6-10 /HPF	0-2 /HPF
Red blood cells	3-5 /HPF	0-2 /HPF
Bacteria	Negative	Negative
Lactic acid	3.0 mmol/L	≤2.0 mmol/L
C-reactive protein	>350 mg/L	≤5.0 mg/L
Human immunodeficiency virus antigen/antibody screen	Negative	Negative
Autoimmune panel	Negative	Negative
Rapid plasma reagin	Negative	Negative

CT scan of the abdomen and pelvis revealed a prostatic abscess, as shown in Figure 1.

**Figure 1:**
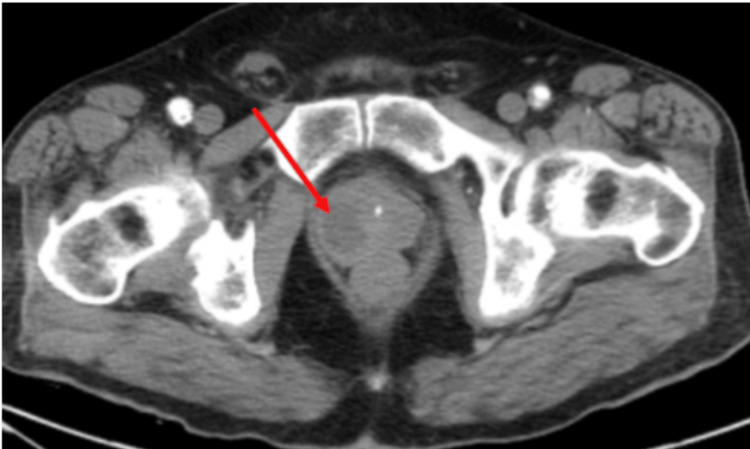
CT of the abdomen and pelvis with contrast showing a cystic structure on the right side of the prostate gland measuring approximately 31 × 24 mm suggestive of a prostatic abscess CT: computed tomography

The patient's blood cultures also showed growth of MSSA. The patient was started on IV cefazolin. An attempt to drain the prostatic abscess by interventional radiology was unsuccessful due to the patient's agitation and clinical deterioration due to atrial fibrillation with a rapid ventricular rate, necessitating transfer to the intensive care unit. The patient was deemed to be a poor surgical candidate for transurethral resection of the prostate due to rapid clinical deterioration.

A CT angiogram of the aorta and iliofemoral arteries raised suspicion for graft site infection (although no erythema or induration was present at the graft site) along with the presence of gas, which was attributed to partial collapse of the stent and vacuum phenomena, thus adding to the diagnostic complexity of this case (Figure 2).

**Figure 2:**
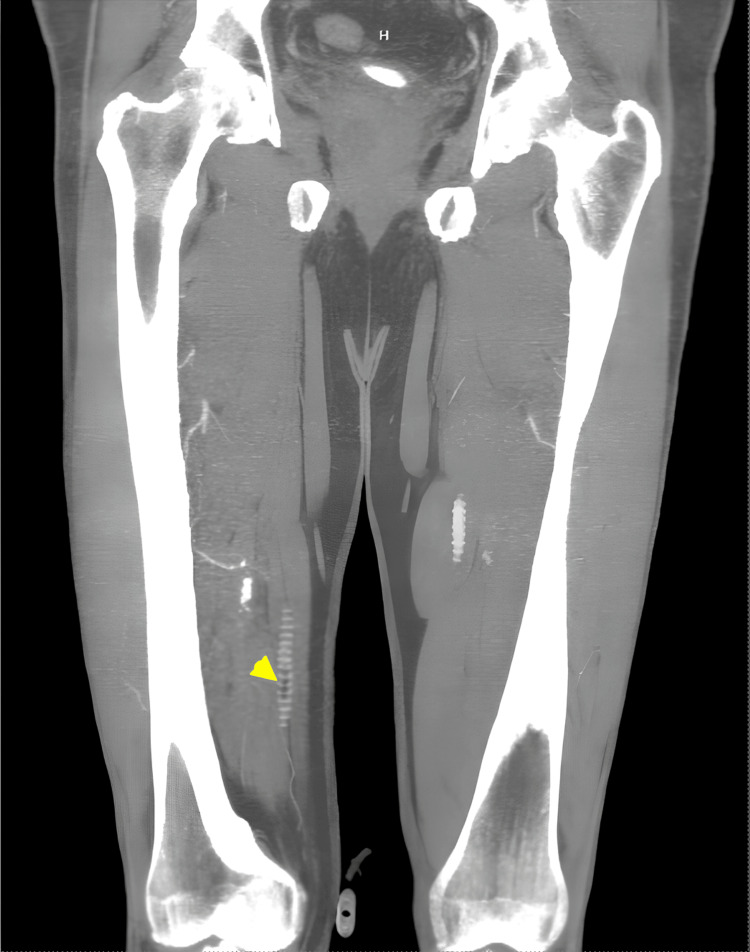
CT angiogram of the aorta and iliofemoral arteries showing some gas (marked by arrowhead) in the right superficial femoral artery with graft occlusion with no surrounding inflammatory changes and may be related to partial collapse of the stent and a vacuum phenomenon CT: computed tomography

A transthoracic echocardiogram was performed, which was unremarkable for any valvular vegetations. A transesophageal echocardiogram could not be performed due to the clinical instability. By that time, the patient started to complain of progressively worsening neck pain, for which a CT scan of the cervical spine revealed an apical abscess at the root of the posterior left mandibular molar, as shown in Figure 3.

**Figure 3:**
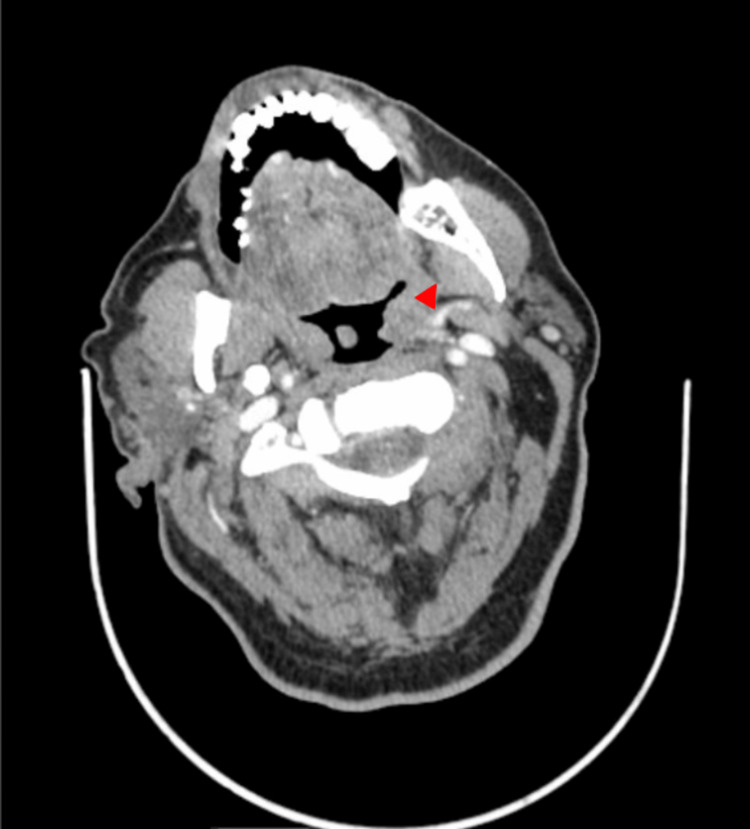
Axial contrast-enhanced CT of the cervical spine, displayed at the level of the oral cavity, demonstrates a hypodense fluid collection (arrowhead) in the left sublingual space, medial to the left mandibular molars, consistent with a left molar abscess CT: computed tomography

CT scan of the lumbar spine revealed heterogeneous enhancement of the kidneys with striated nephrogram, raising suspicion for pyelonephritis as well as multilevel spinal stenosis. Follow-up MRI of the spinal axis revealed a large anterior epidural fluid collection throughout the cervical and thoracic spine, with severe mass effect on the spinal cord and evidence of discitis at the T10-T11 level, as shown in Figure 4.

**Figure 4:**
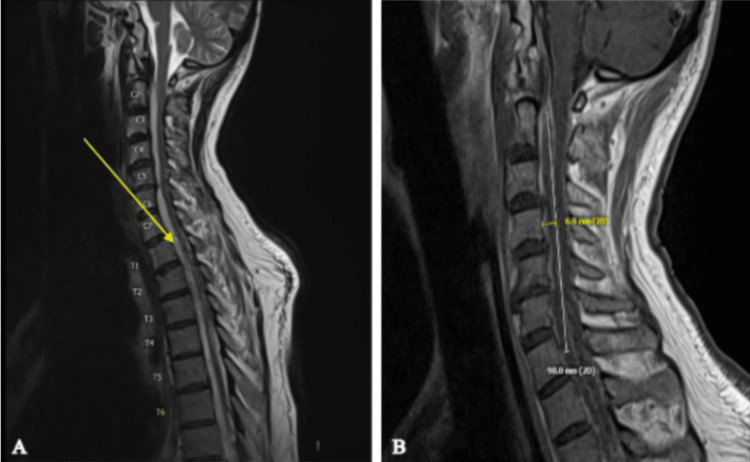
MRI of the thoracic spine with and without contrast in Panel A reveals an extensive anterior epidural abscess from T10–T11 originating from discitis, causing a mass effect on the spinal cord without significant compression. Panel B shows an MRI of the cervical spine with and without contrast, demonstrating a large anterior epidural abscess extending into the thoracic spine with severe mass effect on the spinal cord MRI: magnetic resonance imaging

The patient was emergently taken to the OR by neurosurgery for cervical epidural abscess evacuation and discectomy followed by thoracic epidural abscess evacuation and discectomy. Infectious workup, including multiple sets of blood cultures and intraoperative cultures, continued to show growth of MSSA. IV cefazolin and ertapenem were continued because of their known synergistic activity against MSSA. After extensive neurosurgical procedures, the patient remained intubated, and neurological examination was normal within 24 hours post-neurosurgery when the patient suddenly developed flaccid paralysis in bilateral upper extremities with decreased withdrawal from noxious stimuli in bilateral lower extremities. Repeat MRI of the spinal axis revealed postsurgical changes but no acute findings. MRI of the brain revealed punctate bilateral watershed infarcts, raising suspicion for septic emboli, as shown in Figure 5.

**Figure 5:**
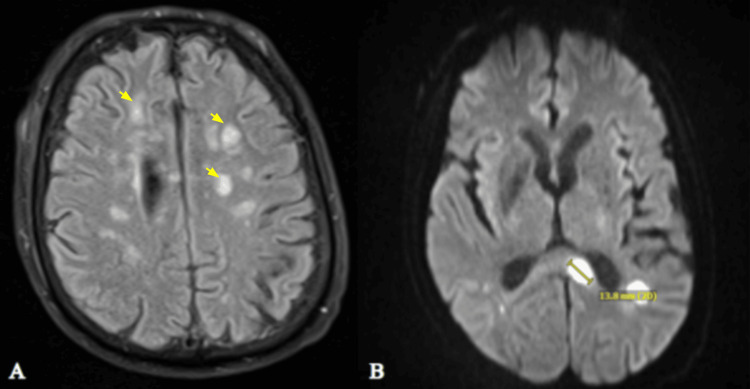
Panels A and B show MRI of the brain demonstrating multiple DWI hyperintensities, suggestive of septic embolic infarcts as marked by arrowheads MRI: magnetic resonance imaging, DWI: diffusion-weighted imaging

At that point, antibiotics were changed to nafcillin, with ertapenem added to improve CNS penetration and for its known synergy with MSSA bacteremia. A high dose of clindamycin was also added for toxin suppression due to concern of toxic shock syndrome leading to rapid clinical decline. The patient subsequently developed worsening renal function, leading to discontinuation of nafcillin. However, renal function continued to decline, and the patient was subsequently started on hemodialysis. His hospital stay was further complicated by the development of ventilator-associated pneumonia with sputum cultures growing *Escherichia coli*, *Pseudomonas*, and *Candida*; he also continued to have intermittent fevers.

Antibiotic coverage further escalated to meropenem, linezolid, micafungin, and rifampin. A repeat brain MRI showed numerous bilateral foci of diffusion-weighted imaging hyperintensity, raising concern for septic embolic infarcts in watershed areas, without evidence of subdural empyema. The patient also developed extensive long-segment left lower-extremity deep vein thrombosis extending from the visualized distal left external iliac vein to the left calf despite being on a direct oral anticoagulant and subsequently started on warfarin; an inferior vena cava filter was placed. Despite aggressive antibiotic treatment, repeated sputum cultures continued to grow *Pseudomonas*. The patient also developed worsening sacral ulcers; wound cultures also grew *Pseudomonas* and *Candida*. The patient subsequently also required sacral ulcer debridement. Goals of care were discussed extensively with the patient's family at the bedside, and they opted for comfort care. The patient subsequently passed away peacefully.

## Discussion

This case illustrates a rare and catastrophic presentation of MSSA bacteremia involving the prostate, epidural space, and CNS, emphasizing the pathogen’s potential for rapid multisystem spread [1,2]. High-risk factors including advanced age, diabetes, peripheral arterial disease, and immunologic vulnerability likely predisposed the patient to invasive infection and poor outcomes [3].

Prostatic abscesses are uncommon, typically caused by *Escherichia coli* or *Klebsiella* [4]; however, MSSA bacteremia can disseminate widely, explaining the patient’s metastatic infections [1,4]. The source of infection remained uncertain, possibly of dental or prostatic origin, with persistent bacteremia due to an inability to drain the abscess, facilitating further spread [5]. A spinal epidural abscess at T10-T11 underscores MSSA’s aggressive hematogenous or venous plexus dissemination [6,7]. Neurological deterioration with flaccid paralysis and bilateral watershed infarcts suggests multifactorial mechanisms, including septic embolization, systemic inflammatory response, and hypoperfusion [7,8].

Suspected vascular graft infection, prostatic abscess, or odontogenic abscess, indicated by CT findings, likely served as a nidus for persistent bacteremia, highlighting the difficulty of establishing the source of infection, distinguishing infection from post-procedural changes, and the importance of surgical evaluation [9]. Renal involvement, evident as heterogeneous enhancement on CT, was attributable to metastatic pyelonephritis, while the limitations of transthoracic echocardiography necessitated consideration of transesophageal echocardiography to detect endocarditis [10-12].

Antimicrobial therapy was strategically tailored. Initial cefazolin, supported by evidence of non-inferiority and a better adverse effect profile, was supplemented with ertapenem to overcome high-inoculum beta-lactamase effects [13]. CNS involvement prompted a switch to nafcillin to ensure adequate CSF penetration, while clindamycin reduced bacterial toxin production in presumed toxic shock syndrome, and rifampin targeted potential prosthetic biofilm formation [13,14].

The patient’s course was complicated by nosocomial infections, acute renal dysfunction, and septic deep vein thromboses, highlighting the challenges of critical care management and pharmacologic limitations in severe sepsis [15]. The development of deep vein thrombosis despite the use of direct oral anticoagulants underscores the prothrombotic milieu in sepsis and the need for careful selection and monitoring of anticoagulation strategies [16].

More broadly, this case reinforces the critical importance of early and effective source control in MSSA bacteremia. A recent comprehensive review demonstrated that in a cohort of 884 patients with *Staphylococcus aureus* bacteremia, shorter time to source control (median one day vs ≥3 days) was associated with earlier clearance of bacteremia and lower mortality, with each additional day of bacteremia conferring a relative risk of death of 1.16 (95% CI, 1.10-1.22; p < 0.001) [17]. In our patient, the inability to achieve adequate source control, secondary to failed percutaneous drainage of the prostatic abscess and clinical instability precluding transurethral resection, likely contributed to persistent bacteremia and the subsequent cascade of metastatic complications. Furthermore, diabetes, a key comorbidity in this patient, has been shown to independently increase the risk of metastatic infection in MSSA bacteremia (OR 2.08; 95% CI, 1.12-3.90), likely through hyperglycemia-mediated impairment of complement activation and neutrophil function [17]. While the findings from this single case cannot establish causality or independently alter clinical practice guidelines, they highlight the importance of aggressive, multidisciplinary source control strategies and heightened vigilance for metastatic foci in diabetic patients with MSSA bacteremia.

## Conclusions

This case emphasizes the need for vigilant source control, including evaluation for silent dental abscesses, early full-spine imaging in the presence of pain, and preference for transesophageal echocardiography when endocarditis cannot be excluded. Multidisciplinary coordination among infectious disease, neurosurgery, and critical care teams is essential to optimize outcomes, especially in patients with multiple comorbidities and limited intervention options. Early recognition, targeted therapy, and proactive management of complications remain critical to interrupting the cascade of catastrophic events in complex MSSA bacteremia.
